# Endoplasmic Reticulum Stress and Autophagy in Homocystinuria Patients with Remethylation Defects

**DOI:** 10.1371/journal.pone.0150357

**Published:** 2016-03-09

**Authors:** Ainhoa Martínez-Pizarro, Lourdes R. Desviat, Magdalena Ugarte, Belén Pérez, Eva Richard

**Affiliations:** Centro de Diagnóstico de Enfermedades Moleculares, Centro de Biología Molecular-SO UAM-CSIC, Universidad Autónoma de Madrid, Campus de Cantoblanco, 28049 Madrid / Centro de Investigación Biomédica en Red de Enfermedades Raras (CIBERER), Madrid, IDIPaz, Spain; University of Hong Kong, HONG KONG

## Abstract

Proper function of endoplasmic reticulum (ER) and mitochondria is crucial for cellular homeostasis, and dysfunction at either site as well as perturbation of mitochondria-associated ER membranes (MAMs) have been linked to neurodegenerative and metabolic diseases. Previously, we have observed an increase in ROS and apoptosis levels in patient-derived fibroblasts with remethylation disorders causing homocystinuria. Here we show increased mRNA and protein levels of Herp, Grp78, IP_3_R1, pPERK, ATF4, CHOP, asparagine synthase and GADD45 in patient-derived fibroblasts suggesting ER stress and calcium perturbations in homocystinuria. In addition, overexpressed MAM-associated proteins (Grp75, σ-1R and Mfn2) were found in these cells that could result in mitochondrial calcium overload and oxidative stress increase. Our results also show an activation of autophagy process and a substantial degradation of altered mitochondria by mitophagy in patient-derived fibroblasts. Moreover, we have observed that autophagy was partially abolished by antioxidants suggesting that ROS participate in this process that may have a protective role. Our findings argue that alterations in Ca^2+^ homeostasis and autophagy may contribute to the development of this metabolic disorder and suggest a therapeutic potential in homocystinuria for agents that stabilize calcium homeostasis and/or restore the proper function of ER-mitochondria communications.

## Introduction

Homocysteine is an amino acid located at a branch-point of metabolic pathways: either it is irreversibly degraded via the transsulphuration pathway to cysteine or it is remethylated back to methionine. Remethylation disorders include defects in methionine synthase (MTR, OMIM ID: 156570), methionine synthase reductase (MTRR, OMIM ID: 602568), MMADHC (OMIM ID: 611935) proteins corresponding to *cblG*, *cblE*, and *cblD-variant 1* cobalamin complementation groups, respectively; and in 5,10-methylene tetrahydrofolate reductase enzyme (MTHFR, OMIM ID: 236250) [1]. Folate derivatives maintain homocysteine at non-toxic levels, via the donation of a carbon group from methyltetrahydrofolate (synthesized by MTHFR) for homocysteine remethylation to methionine. This reaction is catalyzed by MTR that transfers a methyl group from 5-methyltetrahydrofolate to the cob(I)alamin form of the cofactor and from methylcobalamin to homocysteine to form methionine and tetrahydrofolate as products. Optimal activity of MTR requires vitamin B_12_ and MTRR for reductive reactivation of the cobalamin moiety of the vitamin cofactor using S-adenosylmethionine (SAM) as methyl donor to regenerate methylcobalamin. SAM is the primary methyl donor in numerous methylation reactions including DNA methylation and phospholipid biosynthesis [1].

Patients present severe clinical symptoms which are mainly neurological for *MTHFR* deficiency and neurohematological for *MTR* and *MTRR* defects [1]. Hyperhomocysteinemia and defective methionine synthesis are the major pathophysiological mechanisms proposed for these diseases. MTRR knockout mice exhibited short-term memory impairment probably due to methylation disturbances and altered choline metabolism in the hippocampus [2]. In addition, MTHFR deficient mice have been found to have abnormalities in the size and/or structure of the cerebellum, cortex and hippocampus, and to exhibit memory impairment and other behavioral anomalies [3,4].

Several hypotheses have been proposed to explain the pathophysiology of hyperhomocysteinemia, such as alterations in signal transduction pathways, activation of inflammatory factors, oxidative stress, perturbations in calcium homeostasis and endoplasmic reticulum (ER) stress [5]. ER is a unique cellular compartment simultaneously involved in the processes of protein synthesis and Ca^2+^ homeostasis. Various conditions, including oxidative and metabolic stress and Ca^2+^ overload can interfere with ER functions leading to the accumulation of misfolded proteins [6]. Cells respond to ER stress by activating the unfolded protein response (UPR) which consists of three main signaling systems initiated by the stress sensors: PERK, IRE-1 and ATF6. Each pathway activates transcription factors that mediate the induction of a variety of ER stress response genes, such as ATF4, CHOP and XBP1 [7]. All three ER-resident transmembrane proteins are thought to sense ER stress through Grp78 binding/release via their respective luminal domains [7]. Recent studies demonstrated that Herp (Homocysteine-inducible ER stress protein), an ER integral membrane protein, appears to be essential for the resolution of ER stress through maintenance of ER Ca^2+^ homeostasis and for ER-associated protein degradation [6,8]. Upregulation of Herp is important for neuronal survival as Herp knockdown enhances vulnerability to ER stress-induced apoptosis [6].

In addition, ER can connect to and consequently act synergistically with other membranous structures, such as mitochondria. The outer mitochondrial membrane is in contact with a subregion of ER referred to as mitochondria-associated ER membranes (MAMs) which are intracellular lipid rafts that regulate Ca^2+^ homeostasis, metabolism of glucose, phospholipids and cholesterol [9]. Calcium is transferred from ER at MAM by inositol-1,4,5-triphosphate receptors (IP_3_Rs; ER side) which prevents Ca^2+^ accumulation within the ER, and voltage-dependent anion channel (VDAC1; mitochondria side). MAM architecture is complex and involves other proteins, such as: the mitochondrial chaperone Grp75 (glucose-regulated protein 75) that mediates the molecular interaction of VDAC with the IP_3_R allowing a positive regulation of mitochondrial Ca^2+^ uptake [10]; the σ1 receptor (σ-1R) that is a molecular chaperone involved in calcium homeostasis by stabilizing IP_3_R [11]; and mitochondrial dynamin-related fusion protein (Mfn2) that is involved in tethering and regulation of mitochondrial dynamics [12]. ER–mitochondria interface also represents the primary platform for autophagosome formation and the function of pro-survival autophagy machinery by which cells undergo partial autodigestion to briefly prolong their survival under starvation conditions [13]. Autophagy is up-regulated in response to extra- or intra-cellular stress, and defects in this process play significant roles in several human pathologies, including cancer and neurodegeneration [14].

Previously we have observed elevated ROS and apoptosis levels in patients-derived fibroblasts with homocystinuria [15]. The present work was addressed to gain further insight into the contribution of oxidative stress to the pathophysiology of this disorder. With this aim, we focused on the study of ER stress, ER-mitochondria connectivity and autophagy in fibroblasts derived from five patients with homocysteine remethylation disorders.

## Materials and Methods

### Patients and controls´ fibroblasts, culture conditions and reagents

The present study included available fibroblasts from five patients with defects in the *MTRR* (P1, P2, and P3), *MTR* (P4) and *MTHFR* genes (P5); and several control individuals (CC2509 from Lonza (C); and GM08398 (C1), GM08680 (C2), GM08429 (C3) and GM05756 (C4) from Coriell Cell repositories). Patients were referred from clinics to be biochemically and/or genetically diagnosed at Centro de Diagnóstico de Enfermedades Moleculares (CEDEM) in Madrid. Relevant clinical and molecular data about individual patients included in this study are detailed in Table 1. Ethical approval was obtained from the institutional Ethics Committee of the Universidad Autónoma de Madrid for the use of human samples in the present study. The participants provided their written informed consent to participate in this study. Fibroblast cultures were established from patient skin biopsies and maintained with MEM (Minimum Essential Medium) supplemented with 10% fetal bovine serum (FBS), 1% glutamine and 0.1% antibiotic mix (penicillin/streptomycin) under standard cell culture conditions (37°C, 95% relative humidity, 5% CO_2_). Assays were performed when the culture reached 70 to 80% confluence, and control and patients-derived fibroblasts were between passages 7 and 10. Cells were incubated with 2.5 μg/ml tunicamycin (Sigma) for 18 h to activate ER stress, with 1mM trolox (Sigma) for 72 h to scavenge ROS and with 20 mM 3-methyladenine (3-MA, Sigma) for 48 h to inhibit autophagy.

**Table 1:** Genotype and clinical phenotype in five homocystinuria patients with remethylation defects.

Patient no.	Gene affected (OMIM ID)	Allele 1	Allele 2	Onset	Plasma Hcy concentration	Clinical course
P1	*MTRR* (602568)	p.V56M (c.166G>A)	p.V56M (c.166G>A)	EO, 7 m	94 μM	Macrocytic anemia, encephalopathy, myoclonic epilepsy, 25 m, DD
P2	*MTRR* (602568)	p.S454L (c.1361C>T)	p.S454L (c.1361C>T)	EO, 3 m	160 μM	Megaloblastic anemia, 9 m
P3	*MTRR* (602568)	p.G546E (c.1637G>A)	p.R525X (c.1573C>T)	NA	NA	NA
P4	*MTR* (156570)	p.S450D (c.1348_1349TC>GA)	p.V1002_G1003insG (c.3008-4A>G)	EO, 2 m	78 μM	Hypotonia, apnea, brain atrophy, 4 y, DD
P5	*MTHFR* (236250)	p.V450_K510del (c.1530G>A)	p.V450_K510del (c.1530G>A)	EO, 21 d	35 μM	Encephalopathy, metabolic acidosis, DD

EO, early onset (less than 1 year of age); P1, P2, P4 and P5 are alive; DD, developmental delay; NA, not available; y, year; m, month; d, day; P, patient; Hcy, homocysteine.

### Western blot analysis

Adherent cells were harvested from plates by trypsinization in 1X PBS and pelleted by centrifugation for 5 min at 1,500 rpm. Pellets were resuspended in 100 μl of lysis buffer (1% Triton, 10% glycerol, 150 mM NaCl, 10 mM Tris HCl pH 7.5) with protease inhibitors (Roche Diagnostic). Cells were lysed by freezing with liquid nitrogen and thawing at 37°C three times. After centrifugation, protein content from supernatants was determined by the Bradford method (Bio-Rad). Samples were prepared with *NuPAGE*^*®*^ *LDS Sample Buffer* (Invitrogen), dithiothreitol (DTT) and heated 5 min at 100°C. Same amount of protein (50 μg) was run by electrophoresis in 4–12% *NuPAGE*^*®*^ *Bis-Tris Precast Gels* (Invitrogen) at a constant voltage of 120V. *Novex*^*®*^ *Sharp Pre-Stained Protein Standard* (Invitrogen) was used as a molecular weight marker.

Proteins were transferred to nitrocellulose membranes using the *iblot*^®^ *Dry Blotting System* (Invitrogen) device. Membranes were blocked for at least 2 h with blocking solution (1X PBS, 5% low fat milk, 0.05% Tween). Membranes were incubated overnight with the first antibody at its corresponding concentration in blocking solution: 1/5,000 polyclonal anti-Herp (PW9705, Enzo Life Sciences), 1/1,000 polyclonal anti-IP_3_R1 (071210, Millipore), 1/5,000 polyclonal anti-Grp78 (NBP1-06274, Novus Biologicals), 1/5,000 monoclonal anti-Hsp60 (SPA-829, Stressgen Bioreagents), 1/500 monoclonal anti-phospho-PERK (3179, Cell Signaling), 1/1,000 monoclonal anti-Grp75 (Ab171089, Abcam), 1/1,000 monoclonal anti-Sigma1 Receptor (sc-166392, Santa Cruz Biotechnology), 1/1,000 monoclonal anti-Mitofusin 2 (sc-100560, Santa Cruz Biotechnology), 1/1,000 monoclonal anti-LAMP1 (3243, Cell Signalling), and 1/5,000 monoclonal anti-GAPDH (Ab8245, Abcam). Respective secondary antibodies (1/5,000) were hybridized for 1 h at room temperature. Proteins were detected by Enhanced Chemiluminiscence System (GE Healthcare). Quantification was carried out by laser densitometry in a *Bio-Rad GS710 Calibrated Imaging Densitometer* (Bio-Rad), using the software *Quantity One 4*.*3*.*1* (Bio-Rad).

### shRNA and lentivirus infection

Lentivirus incorporating shRNAs were generated in HEK293T packaging cells by cotransfection with pLKO.1 plasmid containing shRNA sequences (Sigma–Aldrich), packing plasmid pCMV-dR8.74 and envelope plasmid pMD2.G (both kindly provided by Dr. J. Diaz-Nido, UAM, Madrid, Spain) using lipofectamine and Plus reagent (Invitrogen). Medium containing viral particles (nontarget control shRNA (SHC002) or either of the *HERP* shRNAs targeting different sequences of human HERP) was harvested at 48 h after transfection and used to infect patients´ fibroblasts with 4 mg/ml polybrene (Sigma–Aldrich). Successfully infected cells were selected and maintained with 1 mg/ml puromycin (Sigma–Aldrich).

### Real time PCR

Total RNA was isolated from control and patients-derived fibroblasts using MagNA Pure Compact RNA Isolation kit and MagNA Pure Compact instrument (Roche Applied Science, Mannheim, Germany). Samples were quantitated spectrophotometrically at 260nm using the Nanodrop ND-1000 photometer (Thermo Scientific, Wilmington, MA). The optimal design of the real-time PCR primers and the Universal ProbeLibrary probe selection was performed using ProbeFinder software (Roche Applied Science). RT-PCR was performed using GeneAmpPCRSystem9700 (Applied Biosystems, Carlsbad, CA), and real-time PCR was performed using LightCycler 480 (Roche Applied Science), both from Unidad de Genómica, Parque Científico de Madrid, UAM, Madrid, Spain. Amplification efficiency and sample-to-sample variation were normalized by monitoring GAPDH.

### RT-PCR

Total RNA was isolated from cultured skin fibroblasts by TRIzol reagent (Life Technologies). First-strand cDNA was synthesized from total RNA (1 μg) using NZY First-Strand cDNA Synthesis Kit (NZYTech), according to the instructions of the manufacturer. Fragments were amplified by PCR using FastStart Taq DNA Polymerase (Roche) and the following primers: XBP1 sense (5´TTACGAGAGAAAACTCATGGC3´), XBP1 antisense (5´GGGTCCAAGTTGTCCAGAATGC3´), GADPH sense (5´GTCGGAGTCAACGGATTTGG3´) and GAPDH antisense (5´TGAGCCCCAGCCTTCTCC3´).

### Cytosolic Ca^2+^ imaging

Fibroblasts from the five patients and one control were plated at a density of 3x10^4^ cells on 12 mm glass cover slips disposed into P24 plates the day before the experiment. Cells were loaded with 5 μM Fura-2AM (Invitrogen) and 0.06% pluronic acid F.127 (Invitrogen) for 30 min at 37°C in Ca^2+^-free HCSS (2.5 mM glucose, 120 mM NaCl, 0.8 mM MgCl_2_, 25 mM Hepes, 5.4 mM KCl, pH 7.4), and washed for 30 min in HCSS (2 mM CaCl_2_, 2.5 mM glucose, 120 mM NaCl, 0.8 mM MgCl_2_, 25 mM Hepes, 5.4 mM KCl, pH 7.4). Then, cover slips were placed into a heated chamber mounted on the microscope stage equipped and Fura-2 fluorescence was imaged ratiometrically using alternate excitation at 340 and 380 nm, and a 510 nm emission filter with a Neofluar 20X/0.75 objective in an Axiovert 75M inverted fluorescence microscope (Zeiss). For single-cell analysis of [Ca^2+^]_i_ the fluorescence ratio intensity at 340 nm (F_(340)_) and 380 nm (F_(380)_) (F_(340)_/ F_(380)_) was obtained. Bradykinin (10 μM; Sigma) and thapsigargin (1 μM; Sigma) were added to induce Ca^2+^ release of ER in the absence of extracellular Ca^2+^. Image acquisition was performed with the Aquacosmos 2.5 software (Hamamatsu).

### Expression of fusion protein LC3B-GFP

Fibroblasts were plated at a density of 2x10^5^ cells on plates *P35G-1*.*5-20-C Sleeve* (MatTek Corporation) and were transfected with *pAutophagSENSE*^*TM*^ *Vector* (Clontech) which contains human LC3B-GFP using *Turbofect*^*TM*^ *transfection Reagent* (Thermo Scientific). After 48 h, autophagosomes were observed with an inverted microscope (Axiovert200, Zeiss) with the fluorescent filters GFP and Texas-Red at 63X magnifications. Images were analysed using Fiji program to determine the number of positive fluorescence points in cells: LC3B+ dots/mm^2^ (autophagosomes/area).

### Immunofluorescence microscopy

8x10^4^ fibroblasts were grown on 10 mm diameter glass cover slips for 24 h. Cells were incubated with lysotraker (1 μM; Molecular Probes) in MEM 10% FBS for 15 min at 37°C in the dark. Cells were rinsed once with MEM 10% FBS and once with 1X PBS, fixed in 20% formalin for 20 min at room temperature, permeabilized 5 min in a solution of 0.2% Triton X-100 in 1X PBS and incubated for 1 h in blocking solution (1X PBS, 2% BSA). For immunostaining, glass cover slips were incubated with anti-cytochrome *c* monoclonal antibody (556432, PharMingen, BD Bioscience) diluted 1/500 in blocking solution (1X PBS, 0.2% BSA) for 1 hour at room temperature. Unbound antibody was removed by washing the cover slips with 1X PBS (three times, 5 min). Secondary antibody anti-mouse (Alexa 488, Invitrogen, emission at 519 nm) 1/500 was used in blocking solution for 1 h in dark at room temperature. Unbound antibody was removed by washing the cover slips with 1X PBS (three times, 5 min). Then, cover slips were put on a slide, with the cells facing down to the slide. Samples were observed with an inverted microscope (Axiovert200, Zeiss) with the fluorescent filters GFP and Texas-Red at 63X magnifications the day after set up.

### Electron microscopy

Fibroblasts were fixed for 2 h in culture plates with 4% paraformaldehyde / 2% glutaraldehyde in phosphate buffer Na/Na_2_ 0.1 M pH 7.4 (0.2 M Na_2_HPO_4_ and 0.2 M NaH_2_PO_4_ in bidistilled water). Cells were washed three times in phosphate buffer Na/Na_2_ 0.1 M pH 7.4 for 10 min and then post-fixed with 1% OsO_4_-1% FeCk for 1 h at 4°C in dark. After dehydration in increasing concentrations of ethanol, 5–10 min for each step: 50, 70, 90, 95 and 100%, impregnation steps and inclusion were performed in Epon and finally polymerized at 60°C for 48 h. 60–80 nm sections were obtained using an ultramicrotome Ultracut E (Leica), and contrasted with uranyl acetate and lead citrate. Observations were performed on a transmission electron microscopy JEM1010 (Jeol) connect to a camera 4Kx4K TemCam-F416 (TVIPS) at 12,000X magnifications.

### ROS and apoptosis assays

Intracellular ROS and apoptosis levels were detected and quantitated by flow cytometry in fibroblasts as described [16].

### Statistical analysis

All results are expressed as mean ± standard deviation, unless stated otherwise. The unpaired Student´s *t* test was used to evaluate the significance of differences between groups. *P* values below 0.05 were considered significant.

## Results

### Analysis of ER stress

**Fig 1:**
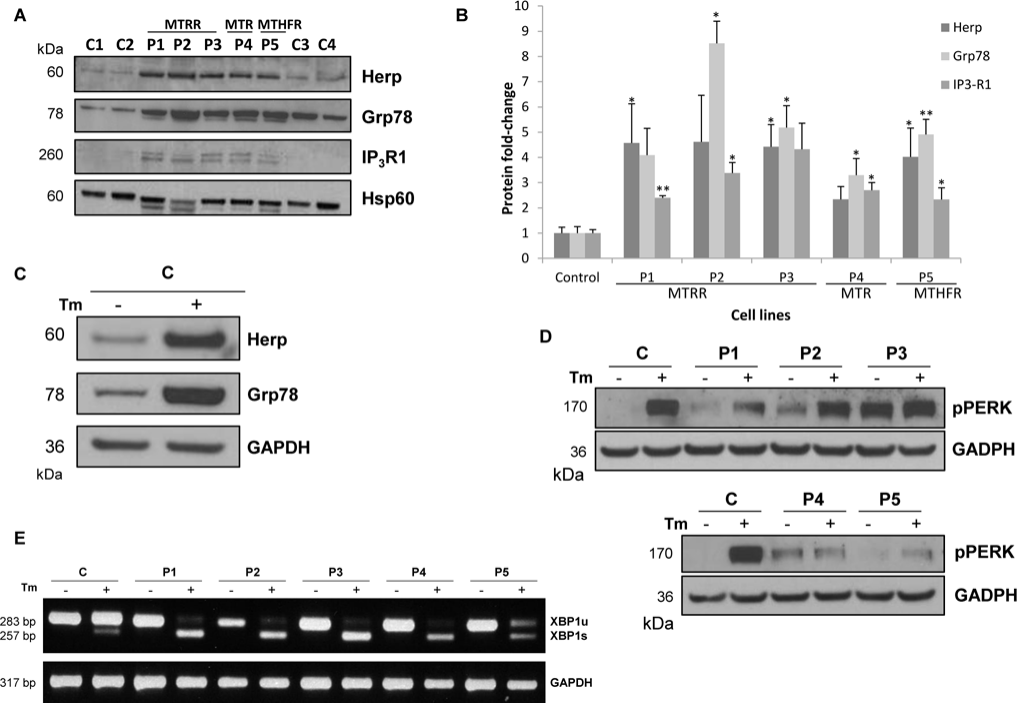
Analysis of protein levels involved in ER stress and Ca^2+^ homeostasis and processing of mRNA XBP1 in control and patients-derived fibroblasts. (A) Equal amounts from controls and patients were loaded (50 μg of total cell lysates) and subjected to Western Blot with anti-Herp, anti-Grp78 and anti-IP_3_R1 antibodies. We used anti-Hsp60 antibody to ensure equal amounts of protein loaded in each lane. This result is representative of three independent experiments. Protein quantification was performed by laser densitometry. The ratios between proteins/Hsp60 for each cell line were calculated to determine the expression fold-change relative to control. (B) Data represent mean ± standard deviation of three independent experiments. (C) Equal amounts from controls were loaded (50 μg of total cell lysates) and subjected to Western Blot with anti-Herp and anti-Grp78 antibodies. We used anti-GAPDH antibody to ensure equal amounts of protein loaded in each lane. This result is representative of two independent experiments. (D) Equal amounts from control and patients were loaded (50 μg of total cell lysates) and subjected to Western Blot with anti-phospho-PERK antibody. We used anti-GAPDH antibody to ensure equal amounts of protein loaded in each lane. This result is representative of two independent experiments. (E) RT-PCR analysis of the processing of mRNA XBP1 transcription factor. Tm: tunicamycin; u: XBP1 unspliced form; s: XBP1 spliced form.

To investigate ER stress in homocystinuria patients with remethylation defects, we have analysed the expression of genes involved in UPR (Grp78, PERK, XBP1, ATF4 and CHOP) and Ca^2+^ homeostasis (Herp and IP_3_R1) at protein or mRNA levels in controls and patients-derived fibroblasts. Immunoblotting results have shown that patients´ cells present significant increased levels of Herp, Grp78 and IP_3_R1 proteins (~2-4-fold) in comparison with control cells (Fig 1A and 1B). It is interesting to note that a degree of variance of Grp78 expression among the normal controls was observed. Induction of Grp78 and Herp expression in normal control fibroblasts treated with tunicamycin was used as positive control for the ER stress induction (Fig 1C). Immunoblotting of phospho-PERK showed an increased protein level in patients-derived fibroblasts compared to control at basal levels (Fig 1D). We have also analysed the processing of mRNA encoding the transcription factor XBP1 in control and patients-derived fibroblasts. Removal of the UPR intron in XBP1 causes a frame-shift, producing an XBP1 protein that is a more potent transcription factor, an event dependent on activation of the IRE1 endoribonuclease domain. RT-PCR results showed an increase in the spliced form of XBP1 mRNA in patients-derived fibroblasts incubated with tunicamycin compared to the control, however, the spliced form was not observed at basal levels (Fig 1E).

**Fig 2:**
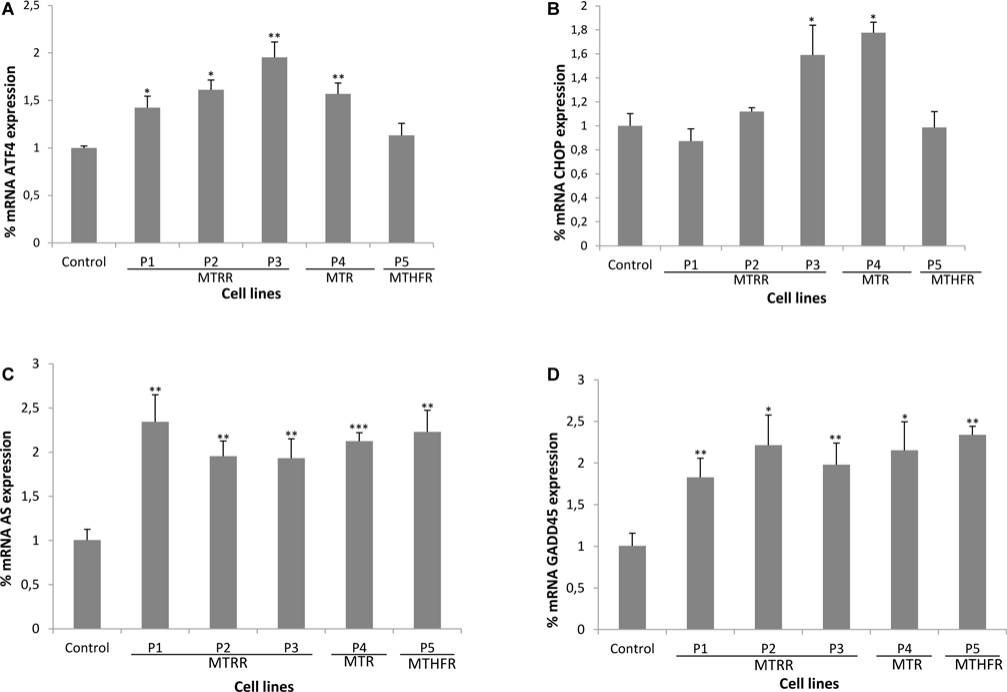
Quantitative gene expression analysis of ATF4, CHOP, asparagine synthase and GADD45 by qRT-PCR. (A, B, C and D) Quantities are shown relative to the expression levels of ATF4, CHOP, asparagine synthase and GADD45 mRNA in control sample. The data represent mean ± SD of three different experiments. *P<0.05; **P<0.01, ***P<0.001.

We next examined mRNA expression of ATF4 and CHOP by qRT-PCR and the results showed an increase in the expression of ATF4 mRNA in P1, P2, P3 and P4 fibroblasts (~1.5-2-fold, Fig 2A) and in the expression of CHOP mRNA in P3 and P4 cells (~1.5-2-fold) compared to controls (Fig 2B). Asparagine synthase (AS) gene is induced in response to amino acid deprivation or ER stress [17,18]. In Tg-I278TCbs^-/-^ (a Cystathionine β-synthase (CBS) mouse model which exhibit extreme hyperhomocysteinemia) the expression of AS and GADD45 genes are induced [19]. qRT-PCR experiments were also performed to determine relative expression of these genes in patients-derived fibroblasts with remethylation defects. Patients´cells showed increased expression of both genes compared to control cells (~2-fold, Fig 2C and 2D).

**Fig 3:**
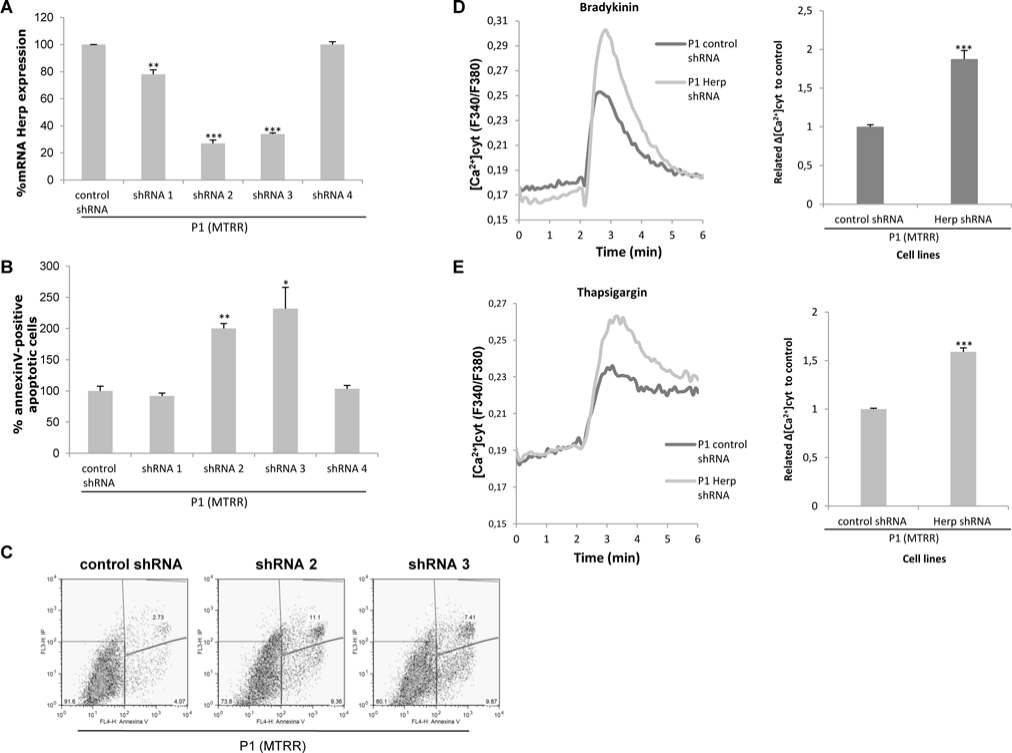
Analysis of mRNA expression, apoptosis and intracellular Ca^2+^ levels after *HERP* gene silencing in P1 fibroblasts. (A) Quantification of Herp mRNA expression by qRT-PCR. Quantities are shown as % Herp mRNA expression relative to fibroblasts infected with non-target control shRNA in P1 cells. Data represent mean ± standard deviation of three independent experiments. (B) Apoptosis detection by flow cytometry. Percentage of annexin V-positive apoptotic cells in Herp shRNAs and non-target control shRNA relative to total cell number per sample. Data represent mean ± standard deviation of two independent experiments, each performed by triplicate. shRNA1, shRNA2, shRNA3 and shRNA4 indicate Herp shRNAs targeting different sequences of human Herp used in this study for the generation of lentiviral particles and infection of fibroblasts. (C) Dot blots from a representative experiment of apoptosis levels in P1 fibroblasts infected with Herp shRNA2 and shRNA3 and control shRNA are shown. (D) Representative recording showing levels of intracellular Ca^2+^ before and after addition of 10 μM bradykinin (BK) in control shRNA and knockdown Herp P1 cells. Results were obtained from two independent experiments. Data represent mean ± SEM (standard error of the mean = SD/$\sqrt{21}$). **(**E) Representative recording showing levels of intracellular Ca^2+^ prior to and after exposure of control shRNA and knockdown Herp P1 cells to 1 μM thapsigargin (Thap). Results were obtained from two independent experiments. Data represent mean ± SEM (standard error of the mean = SD/$\sqrt{21}$). *P<0.05; **P<0.01; ***P<0.001.

Herp appears to play an essential role in preventing death and in stabilizing cellular Ca^2+^ homeostasis in neuronal cells subjected to ER stress [6]. We have examined apoptosis and calcium levels in Herp-silenced fibroblasts with homocystinuria to test this protector role. First, we analysed the apoptosis rate in P1 patients´cells treated with shRNAs targeting Herp. qRT-PCR was performed to confirm gene-silencing effects at the mRNA levels. The different Herp shRNAs resulted in reductions of 20–25% for shRNA1, 60–75% for shRNA2, 60–65% for shRNA3 and 25% for shRNA4 (Fig 3A). To determine if *HERP* gene silencing has an effect in apoptosis in homocystinuria, we next analysed the percentage of annexinV-positive apoptotic cells in Herp shRNA and non-target control shRNA cells by flow cytometry. Interestingly, the results showed that apoptosis levels were increased in those shRNA cells in which Herp mRNA expression is highly reduced (shRNA2 and shRNA3). shRNA2 and shRNA3 fibroblasts showed a ~2-fold increase in apoptosis in P1 compared to control shRNA cells (Fig 3B and 3C).

To test the stabilization role of Herp protein in ER Ca^2+^ homeostasis, we analysed calcium levels in P1 fibroblasts treated with Herp shRNA2 and control shRNA. Knockdown of Herp substantially increased the amplitude of the bradykinin (BK)-induced Ca^2+^ transients (Fig 3D) that activates phospholipase C and subsequently releases IP_3_ to release internal calcium stores. BK evokes a rapid and transient increase in cytosolic Ca^2+^, the amplitude was significantly increased in Herp silenced cells compared to the non-target control shRNA in P1 fibroblasts (~2-fold) (Fig 3D). The ER Ca^2+^ store was measured as the rapid increase in intracellular Ca^2+^ after the addition of thapsigargin to the cells, an inhibitor of SERCA pump that blocks calcium uptake into ER, causing the diffusion of calcium from ER to the cytosol due to a very strong calcium gradient. The peak cytosolic Ca^2+^ elevation induced by thapsigargin was higher in Herp silenced P1 fibroblasts compared to non-target control shRNA (~1.5-fold) (Fig 3E).

### Analysis of ER-mitochondria contacts and calcium levels

**Fig 4:**
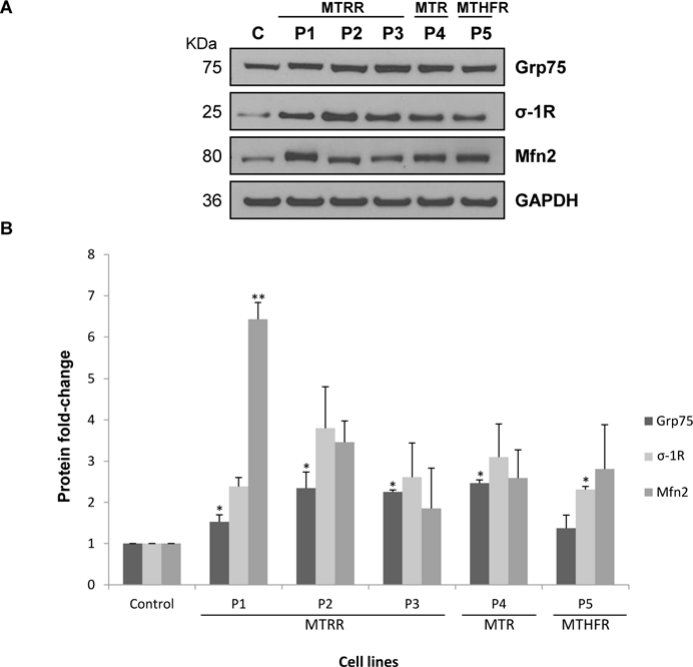
Analysis of MAM-associated protein levels in control and patients´ fibroblasts. (A) Equal amounts from one control and the five patients were loaded (50 μg of total cell lysates) and subjected to Western Blot with anti-Grp75, anti-σ-1R and anti-Mfn2 antibodies. We used anti-GAPDH antibody to ensure equal amounts of protein loaded in each lane. This result is representative of three independent experiments. (B) Protein quantification was performed by laser densitometry. The ratios between proteins/GAPDH for each cell line were calculated to determine the expression fold-change relative to control. Data represent mean ± standard deviation of three independent experiments (*P<0.05; **P<0.01).

Since MAM-associated proteins play an important role in calcium shuttling, we have analysed the expression of Grp75, σ-1R and Mfn2 in fibroblasts from patients with homocystinuria by immunoblotting. We observed increased levels of Grp75 (1.5–2.5-fold), σ-1R (2.5-4-fold) and Mfn2 (2–6.5-fold) proteins in patients-derived fibroblasts compared to control (Fig 4A and 4B).

**Fig 5:**
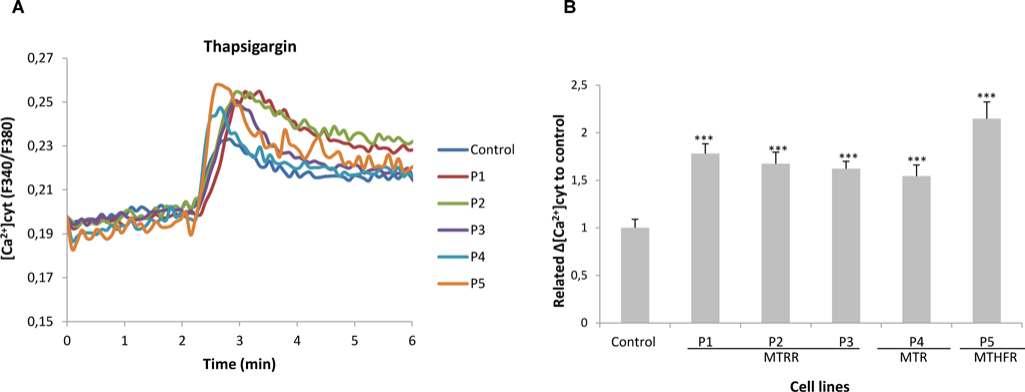
Levels of intracellular Ca^2+^ in control and patients´ fibroblasts. (A) Representative recording showing levels of intracellular Ca^2+^ prior to and after exposure of patients and control to 1 μM thapsigargin (Thap). (B) Quantitative data from three different experiments showing the peak amplitude of the Thap-induced rise in cytosolic Ca^2+^ in patients and control fibroblasts. Data represent mean ± SEM (standard error of the mean = SD/$\sqrt{40}$) (***P<0.001).

ER stress has previously been shown to influence ER-mitochondria coupling and their Ca^2+^ cross-talk [20]. To detect changes in Ca^2+^ shuttling from ER to mitochondria in homocystinuria patients in basal conditions, we analysed the calcium responses by applying thapsigargin. The peak amplitude of thapsigargin-evoked calcium gradient was increased in all patients´ cells (~1.5-2-fold) (Fig 5A and 5B).

### Analysis of autophagy process

**Fig 6:**
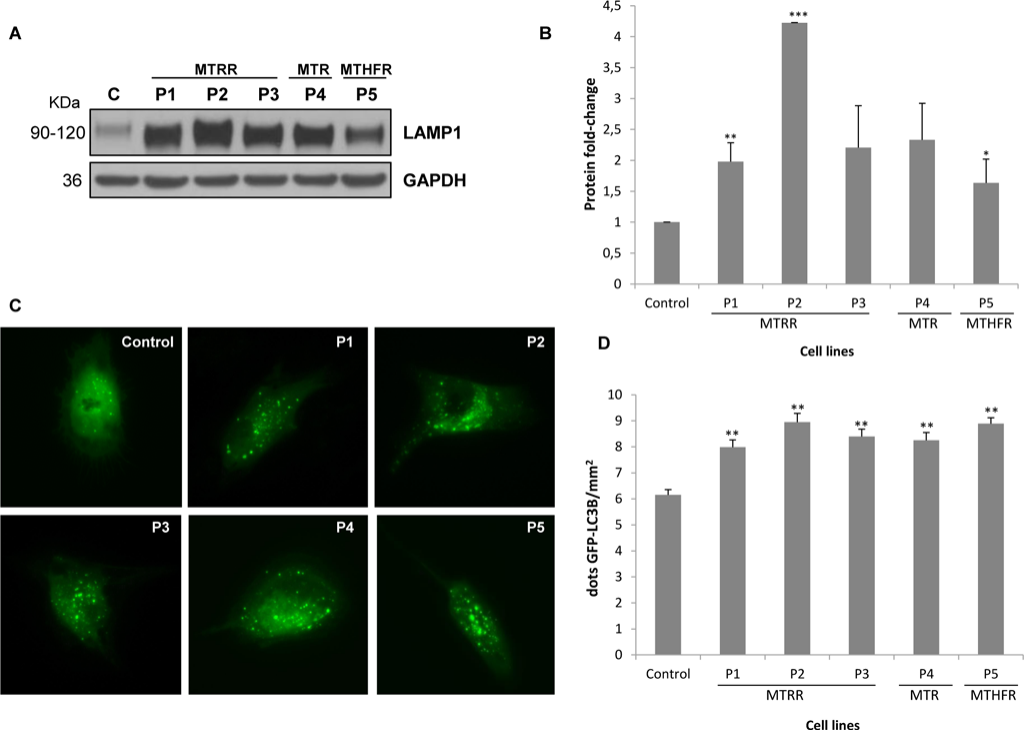
Analysis of autophagy process in patients-derived fibroblasts with homocystinuria. (A) Analysis of protein levels by immunoblotting. Equal amounts from control and the five patients were loaded (50 μg of total cell lysates) and subjected to Western Blot with anti-LAMP1 antibody. We used anti-GAPDH antibody to ensure equal amounts of protein loaded in each lane. This result is representative of three independent experiments. (B) Protein quantification was performed by laser densitometry. The ratios between proteins/GAPDH for each cell line were calculated to determine the expression fold-change relative to control. Data represent mean ± standard deviation of three independent experiments. (C) Analysis of autophagosomes formation by fluorescence microscopy. Representative images of the autophagosome monitorization in control and patients-derived fibroblasts. (D) Quantification of autophagosome number per area in patients´cells compared to control. Results were obtained from three independent experiments. Data represent mean ± SEM (standard error of the mean = SD/$\sqrt{80}$) of positive fluorescence points of LC3B in each mm^2^ of 80 cells analysis. *P<0.05; **P<0.01; ***P<0.001.

Our previous results showed increased ROS and apoptosis levels in patients-derived fibroblasts with homocystinuria [15]. Because changes in cell death by autophagy merit further investigation with respect to homocystinuria, we analysed protein levels of a specific autophagy marker: LAMP1 (lysosomal-associated membrane protein 1), as well as autophagosome formation in live cells. Protein levels of LAMP1 were significantly increased in all patients´ cells compared to control (~2-4- fold, Fig 6A and 6B).

Next, autophagy was monitored by following the redistribution of a green fluorescent GFP-LC3B fusion protein from the cytosol to the forming autophagosomes by microscopy. We observed an increased number of autophagosomes/area in all patients´ fibroblasts compared to controls (Fig 6C and 6D).

**Fig 7:**
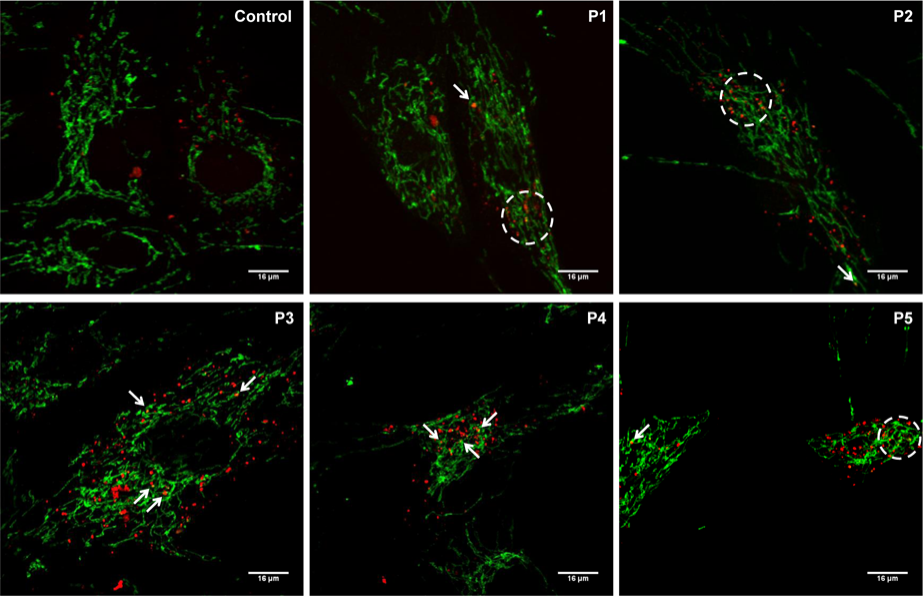
Mitochondrial degradation during autophagy in control and patients´ fibroblasts by immunofluorescence analysis. Cultured fibroblasts were incubated with lysotraker (lysosomal marker; red); inmunostained with anti-cytochrome *c* (mitochondrial marker; green) and examined in a fluorescence microscope (63X magnifications) as described in Material and Methods. Colocalization of both markers was assessed by Fiji program (Bar = 16 μm).

To confirm the presence of mitochondria degradation or mitophagy in homocystinuria cells; we performed a double staining immunofluorescence of cytochrome c (mitochondrial marker) and lysotraker (lysosomal marker) in control and patients´ fibroblasts. Fig 7 shows that cytocrome c colocalized with lysotraker (arrows and circles) in all patients-derived fibroblasts in comparison with the control where the colocalization was not observed, suggesting that mitochondria were being degraded within autophagosomes in patients´ cells.

**Fig 8:**
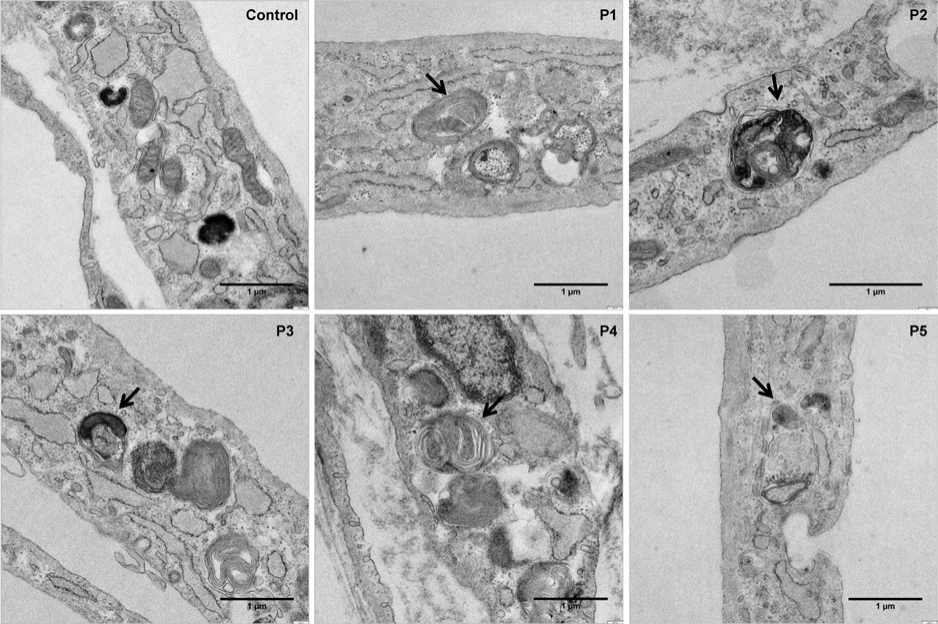
Mitochondrial ultrastructure analysis. Representative electron micrographs of control- and patients-derived fibroblasts. Control fibroblasts showed mitochondria with typical ultrastructure. Laminar bodies and autophagosomes with mitochondria were observed in patients´ fibroblasts. (Bar = 1μm).

To verify this observation we also analysed mitochondrial ultrastructures in control and patients´ fibroblasts by electron microscopy. Patients´ cells exhibited a reduced number of mitochondria and a higher presence of laminar bodies (lysosomes and autophagosomes) compared to control indicating extensive autophagy. In addition, the results clearly showed that mitochondria are being degraded in early autophagosomes (Fig 8).

**Fig 9:**
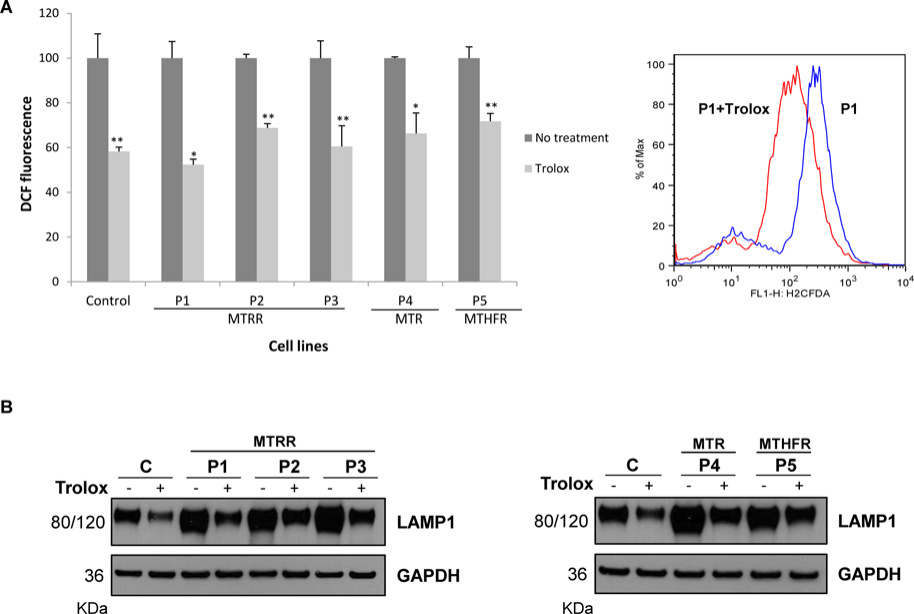
Analysis of trolox effect in ROS levels and autophagy marker´s levels in control and patients´ fibroblasts. (A) ROS content was determined by flow cytometry using H_2_DCFDA as fluorescence probe after cells were incubated for 72 h with 1 mM of trolox. Results are expressed as DCF fluorescence relative to untreated cells. Data represent mean values ± standard deviation from two experiments performed by triplicate (*P<0.05; **P<0.01). A representative overlap of ROS levels in untreated P1 cells and trolox-treated P1 fibroblasts is shown. (B) Cells were incubated with 1mM of trolox for 72 h. Equal amounts of protein from control and the five patients´ fibroblasts were loaded (50 μg of total cell lysates) and subjected to immunoblotting with anti-LAMP1 antibody. We used anti-GAPDH antibody to ensure equal amounts of protein loaded in each lane. This result is representative of three independent experiments.

To further examine the role of ROS generation in autophagy, we cultured patients´ fibroblasts in the presence of the antioxidant trolox (a vitamin E analogue that inhibit the propagation phase of the peroxidative process by neutralizing the lipid-derived radicals) and analysed the levels of LAMP1 protein by Western Blot. Results showed that trolox caused a 30–50% reduction in ROS levels in fibroblasts from control and all patients (Fig 9A); and that the decrease of ROS levels attenuated the expression of LAMP1 protein (Fig 9B).

**Fig 10:**
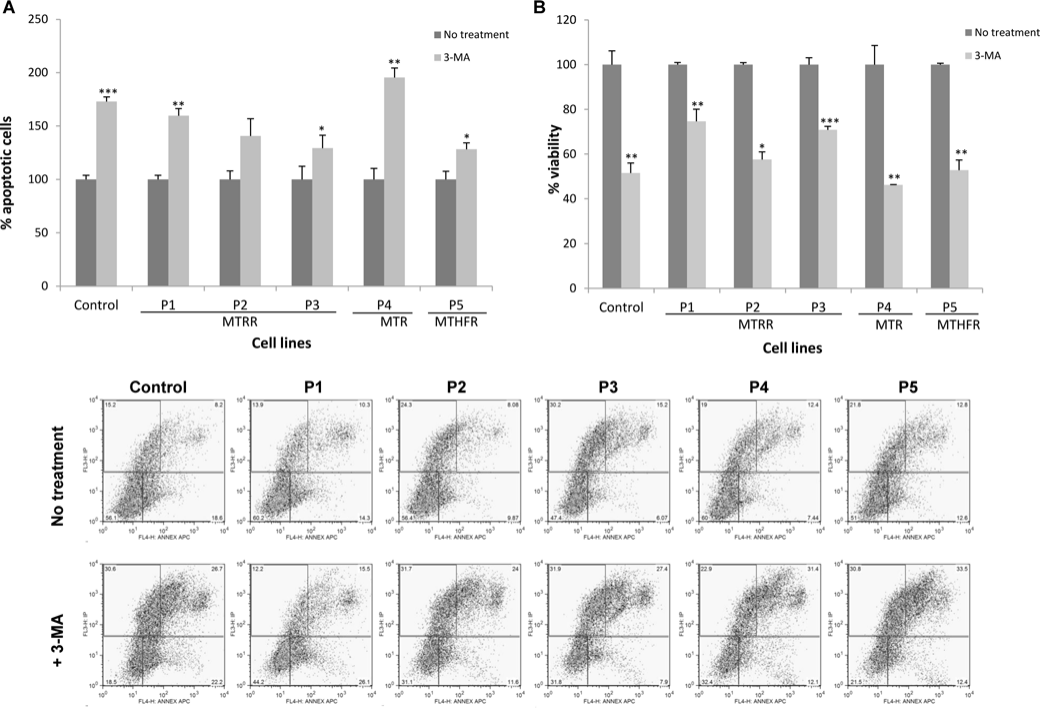
Autophagy inhibition and analysis of apoptosis in control and patients´ fibroblasts by flow cytometry. Cells were incubated for 48h with 20 mM of 3-MA. Data represent mean values ± standard deviation from two experiments performed by triplicate (*P<0.05; **P<0.01; ***P<0.001). (A) Percentage of annexinV-positive apoptotic cells in patients and control cells relative to total cell number per sample. (B) Percentage of viability in patients and control cells relative to total cell number per sample. Dot blots from a representative experiment of apoptosis levels are shown.

In order to elucidate whether autophagy was a protective or pathological mechanism, we examined the effect of blocking this process using 3-methyladenine (3-MA, an inhibitor of type III phosphatidylinositol 3 kinases blocking autophagosome formation) and evaluated the viability and apoptosis in cells by flow cytometry. The results clearly showed that the autophagy inhibition significantly reduces cell viability and increases the rate of apoptosis (Fig 10A and 10B). These observations suggest that autophagy may play a protective role through the recycling/elimination of dysfunctional mitochondria.

## Discussion

The elevation of homocysteine has been correlated with complex and multifactorial diseases, including cardiovascular diseases, neurodegenerative diseases, and neural tube defects; and its toxic effects have been frequently attributed to direct or indirect perturbation of redox homeostasis [5]. Since homocystinuria patients-derived fibroblasts with remethylation defects previously showed an increase in ROS and apoptosis levels [15], this work aims to deepen our understanding of ROS effects at the cellular level analysing ER stress, ER-mitochondria connectivity and autophagy.

Several mechanisms have been proposed to explain the pathological changes associated with hyperhomocysteinemia. It has been reported that homocysteine causes protein misfolding in the ER and activates the UPR, leading to increased expression of the ER stress-response genes [21]. However, other studies not supporting this hypothesis should also be considered. A mouse model of HCU (CBS-deficient homocystinuria) did not exhibit hepatopathy due to the induction of ER stress by homocysteine probably due to the protective effects of cystathionine [22]. Increased growth of the brain in patients with MTHFR deficiency has been observed with early betaine therapy which did not normalize plasma total homocysteine [23]. In addition, Mthfr^-/-^ mouse has a 10-fold elevation in plasma homocysteine (30 μM) and does not exhibit oxidative or ER stress [24,25].

Numerous proteins are regulated by ER stress to generate homeostatic and protective responses. Our results point to the presence of ER stress in patients-derived fibroblasts with homocystinuria. These cells showed increased levels of several proteins involved in the ER stress response like ATF4, pPERK, CHOP, Grp78, AS, GADD45 and the homocysteine-inducible ER stress protein Herp. The induction of these ER stress response genes has previously been revealed by mRNA differential display and cDNA microarray analyses when cells were exposed to supraphysiological concentrations of homocysteine [5]. Activation of ER chaperones, such as Grp78, plays an important role in adaptive UPR since it improves protein folding and prevents ER stress-induced apoptosis. ATF4 regulates protein translation during ER stress and stimulates the expression of CHOP, a transcription factor that induces apoptosis through several mechanisms [26]. Interestingly, glutathione (GSH) synthesis is directly coupled to the integrated stress response (IRS), and loss of ATF4 impairs GSH production by inhibiting the expression of cystathionine γ-lyase (CGL) highlighting the link between the IRS and the transsulphuration pathway [27].

The presence of the spliced form of XBP1 mRNA in patients-derived fibroblasts incubated with tunicamycin indicates that fibroblasts from patients are significantly more sensitive to the treatment. However, the spliced form was not observed at basal levels, suggesting non activation of the UPR by the IRE1 branch. The induction of human AS gene following activation of the UPR was previously reported in mammalian cells [18] and in Tg-I278TCbs^-/-^ mouse model in which an increased GADD45 expression was also observed [19]. The induction of the DNA damage response through increased expression of GADD45 could emphasize cellular stress as has been described in fibroblasts from patients with OXPHOS complex I deficiency [28].

It has been reported that during the terminal differentiation of mature B lymphocytes into antibody secreting plasma cells, IRE1 and ATF6, but not PERK, are activated, and further that activation of ATF6 is preceded by activation of IRE1 [29]. The activation of the three UPR sensors seems not to be a concerted process *in vivo* and differences in the abilities of individual UPR initiators to recognize or respond to various forms of ER stress can be observed; the UPR components display distinct sensitivities toward different forms of ER stress [30]. In addition, the UPR in cultures of human fibroblasts is versatile and differs depending on the specific type of ER stress [31]. It is possible that in a disease state these pathways could be also differentially or selectively regulated, and that one or more branches of the UPR are important in the pathogenesis of several diseases. Therefore, these observations could explain the variances observed in the UPR sensors in our patients-derived fibroblasts with remethylation defects. Indeed, PERK branch seems to be activated in our cell model, but that activation is subtle.

There are evidences suggesting that the induction of ER chaperones by UPR is coordinated with a decreased rate of protein synthesis and the G1 cycle arrest [32]. In addition, homocysteine causes ER stress and growth arrest in human umbilical vein endothelial cells [33]. We have observed differences in growth rate of fibroblasts from the different patients compared to the control used in this study. Fibroblasts from P5 and P4 and control grew essentially at equivalent rates. P1 and P3 grew basically at equivalent rates between them but at lower rate compared to the control. Finally, P2 grew more slowly compared to the control and the rest of patients-derived fibroblasts. These differences could be due to a number of factors which are controlling growth rate, such as the ability to make methionine from homocysteine, the methylfolate trap to prevent methyl group deficiency resulting from a very low supply of methionine, ER stress, etc. Indeed, the variability among cell lines may be even explained taking into account other unknown regulatory or epigenetic factors. The alterations observed in growth rate of the cultured cells could be, at least in part, due to the differences in ER stress levels and plasma homocysteine concentration presented in each cell line. It is interesting to note that the level of homocysteine in the MTHFR deficient patient (P5) is very low, and the observed ER stress in P5 fibroblasts is probably not due to this metabolite.

Another important aspect to consider in our cell model is the expression of other methionine/folate/thiol metabolism enzymes. CBS activity in cultured human fibroblasts is detectable [34]. However, reports of the presence of CGL in this type of cell have been conflicting: CGL activity in normal cultured skin fibroblasts has been described in two works [35,36], whereas two other groups found no detectable activity [36]. If we consider that CGL expression in cultured fibroblasts is variable, the amount of cystathionine could be relevant since is capable of protecting against the pathological effects of ER stress without modulating the UPR [22]. Up to date patients-derived fibroblasts have been used, with their limitations, for modeling human disease. So, as with any crude biological assay, these results should be interpreted with caution. Nevertheless, it might provide clues to the disease physiopathology as has been reported for other human diseases [37,38,39].

Protein levels of Herp are markedly increased in neurons subjected to ER stress [6] and in substantia nigra in Parkinson disease [40]. Our present studies also show an increase in cell death after Herp knockdown. We postulate that homocysteine in homocystinuria patients could modify the redox environment of ER and also induce an elevation of Herp protein which has a protective role against death under conditions associated with ER stress as described previously [6]. It is noteworthy that several reports have described the roles of Herp but how Herp contributes to the restoration of ER homeostasis remains unclear. ER stress increases the basal ER Ca^2+^ content, which is associated with marked increase in Ca^2+^ fluxes across the ER membrane, decreased mitochondrial membrane potential and increased vulnerability of the cells to apoptosis [41]. The ability of Herp to prevent ER stress-induced death has been correlated with its ability to stabilize cellular Ca^2+^ [6]. Our observations corroborate this Herp function in regulating total ER Ca^2+^ load and release since bradykinin and thapsigargin evoke an increase in [Ca^2+^] in Herp-silenced patient cells. In addition, our results show increased protein levels of IP_3_R1 suggesting an aberrant accumulation of ER Ca^2+^ channels that could lead to disruption of ER Ca^2+^ homeostasis as described previously in cellular models of neuronal degeneration [42].

The close contact points between ER and mitochondria at specialized regions of ER (MAMs) are important for Ca^2+^ handling, among other cellular and physiological functions [10]. An increased ER-mitochondria connectivity was detected in human fibroblasts from individuals with familial and sporadic Alzheimer Disease and the increased cross-talk between these two organelles has been considered relevant in the pathogenesis of this devastating disease [43]. The up-regulation at protein level of three MAM-associated proteins (Grp75, σ-1R and Mfn2) observed in homocystinuria patients´ cells may indicate an altered ER-mitochondrial communication suggesting an aberrant calcium homeostasis in this disease. The cytosolic Ca^2+^ monitorization using thapsigargin in patients´ cells supports this hypothesis. The increase in cytosolic Ca^2+^ levels observed in all patients after thapsigargin addition indicates that the ER stress-induced perturbation of the intracellular Ca^2+^ level could be explained by a higher release of ER luminal Ca^2+^ which produces a higher depletion of ER Ca^2+^ stores.

The previous observations suggest the presence of calcium perturbations in homocystinuria patients´ cells probably affecting many downstream processes, such as signalling, regulation of biochemical pathways, apoptosis and mitochondrial dynamics which could also be aggravated with the increase in ROS levels previously detected [15]. Recently, it was also shown that MAMs are important for autophagy by regulating autophagosome formation; ER-mitochondria interface provides membranes for autophagy [13]. Our results suggest that autophagy and mitophagy processes could be activated in homocystinuria fibroblasts. Autophagy in homocystinuria patients´ fibroblasts may be characterized by a selective degradation of dysfunctional mitochondria or mitophagy as has been described in CoQ deficient fibroblasts [44] and in neurodegenerative diseases [45,46]. Since the markers used in our work are consumed by the processes, further studies are needed to determine which step is altered in autophagy process in homocystinuria disease.

Moreover, we tested the effect of the ROS scavenger trolox to address if ROS have a role in autophagy and the reduction in LAMP1 levels after antioxidant treatment suggest that ROS generation could be involved in the autophagy process. It is well established that autophagy can protect against neurodegeneration [47]; autophagy can remove damaged mitochondria that would otherwise activate caspases and apoptosis. We demonstrated that disruption of autophagic processing by 3-MA promotes cell death suggesting that autophagy plays a protective role through the recycling/elimination of dysfunctional mitochondria.

Our observations have relevant implications in the pathophysiology of homocystinuria disease. This work is the first to report ER stress, calcium homeostasis deregulation, an altered ER-mitochondria connectivity along with mitophagy in patients-derived fibroblasts. Elucidation of the cellular and molecular mechanisms that promote or prevent disturbances in ER Ca^2+^ homeostasis may lead to novel approaches for therapeutic intervention for this human pathology.
